# Self-Reported Use of Personal Protective Equipment among Emergency Department Nurses, Physicians and Advanced Practice Providers during the 2020 COVID-19 Pandemic

**DOI:** 10.3390/ijerph18137076

**Published:** 2021-07-02

**Authors:** Roslyn M. Seitz, Anna Q. Yaffee, Elizabeth Peacock, Timothy P. Moran, Andrew Pendley, Jonathan D. Rupp

**Affiliations:** Department of Emergency Medicine, Emory University, Atlanta, GA 30322, USA; AYaffee@emory.edu (A.Q.Y.); Elizabeth.Peacock@emory.edu (E.P.); Timothy.Patrick.Moran@emory.edu (T.P.M.); Apendle@emory.edu (A.P.); jonathan.rupp@emory.edu (J.D.R.)

**Keywords:** COVID-19, personal protective equipment, infection prevention, emergency department, health care worker

## Abstract

Background: Emergency departments (EDs) have seen dramatic surges in patients infected with COVID-19 and are high-risk transmission environments. Knowledge, attitudes and practice regarding personal protective equipment (PPE) among ED health care workers (HCWs) during the COVID-19 pandemic have not been studied, thus this study examines this knowledge gap. Methods: This was a cross-sectional survey of 308 HCWs in two urban EDs in Atlanta, Georgia in April and May of 2020. Results: We surveyed 308 HCWs; 137 responded (44% response rate). All HCWs reported adequate knowledge and 96% reported compliance with PPE guidelines. Reported sources of PPE information: 56.7% charge nurse, 67.3% the institutional COVID-19 website. Frequency of training was positively associated with understanding how to protect themselves and patients (OR = 1.7, 95% CI: 1.0–2.9). Conclusions: Few HCWs are willing to care for patients without PPE, and therefore we should aim for resiliency in the PPE supply chain. EDs should consider multiple communication strategies, including a website with concise information and enhanced training for key personnel, particularly the charge nurse. Attention to frequency in HCW training may be key to improve confidence in protecting themselves and patients. Findings can be leveraged by EDs to implement effective PPE training.

## 1. Introduction

The COVID-19 pandemic has led to over 32,623,220 infections in the United States [1] and has brought to the forefront of national attention the challenges with the supply chain of personal protective equipment (PPE). The lack of a resilient supply chain for PPE has left health care workers (HCWs) unprotected from SARS COV-2 transmission. Additionally, at the beginning of the pandemic, frequent changes to recommendations led to significant confusion and inconsistencies regarding infection control and prevention practices. Infection control and prevention practices can improve patient outcomes [2,3], and are paramount to prevent nosocomial spread of infections to HCWs. Unfortunately, even during non-pandemic times, adherence to infection control recommendations is poor [4,5,6,7,8]. Understanding perceptions and barriers of PPE use in emergency departments (EDs) HCWs is key to advancing effective PPE training.

A survey instrument was developed to study HCW knowledge, attitudes and practices of PPE in intensive care units (ICUs) during previous influenza pandemics in the United States and China [9,10]. Utilizing this instrument, the objective of this study was to assess ED HCW knowledge, attitude and institutional factors in response to the COVID-19 pandemic. Our findings can provide understanding of ED HCW perceptions of PPE and can be leveraged by other EDs to help design and implement effective PPE training and communication strategies

## 2. Materials and Methods

This study took place in 2 academic EDs located in a large metropolitan area and staffed by 61 physicians, 53 advanced practice providers (APPs; nurse practitioners and physician assistants) and 194 nurses. All ED staff (308) were eligible for this study with the exception of the study authors. The survey was administered by email between 24 April and 14 May of 2020. Subjects consented to the voluntary study by completing the survey on an online, anonymous and secure platform. This study was approved by the institutional review board. No incentive was offered for participation.

This survey consisted of a 37-question instrument (Supplementary Materials) evaluating participants’ knowledge, attitudes and organizational factors related to PPE use while caring for patients under investigation for COVID-19 (PUIs). To encourage participation in an anonymous fashion, we collected limited demographic information (Table 1).

The original PPE survey was developed by Daugherty et al. in 2009 and was based on published studies of hand hygiene behavior [7,11] and the theory of planned behavior. Thus, the survey contains elements that address (1) behavioral beliefs (beliefs about the impact of the expected behavior); (2) subjective norms (beliefs about the expectations of those in the organization); and (3) perceived behavioral control (beliefs about one’s ability to implement the expected behavior) as they related to PPE use [12]. 

Additional questions were added to the survey to collect data on the number of times respondents received PPE training prior to caring for PUIs and if respondents believed PPE was fairly distributed between personnel. Respondents were asked whether they had suspected or confirmed COVID-19 and if they would be more likely to care for COVID-19 patients if they had a test which told them if they had previous infection (i.e., antibody testing). 

Categorical data were described using frequencies and percentages. Continuous variables were described using medians and interquartile ranges. Analyses were conducted in order to determine whether self-reported compliance with COVID-19 precautions (reported on a 0–100 scale) and the number of times the respondent underwent PPE training were associated with survey answers. Binary logistic regressions were used to evaluate the associations between compliance/training and survey items with binary response options: (1) “I understand the relevant knowledge of safe PPE use”, (2) “Are you willing to treat and/or care for patients with COVID-19 infection if you have the opportunity?”, (3) “Are you willing to treat and/or care for patients with COVID-19 infection if you do not have the recommended PPE?”, and (4) “I would be more likely to care for patients with suspected or confirmed COVID-19 infection if I had a test which told me if I had previously recovered from the infection”. The remaining items used ordinal response options and the associations between compliance/training and those survey items were evaluated with ordinal logistic regressions. Across the dataset, 0.7% of the data were missing, and the pattern of missing data was consistent with missing completely at random mechanisms (Little’s MCAR test *p* = 0.60). Ten complete datasets were imputed using fully conditional specification [13]. Analyses were conducted using SPSS (v.25; Armonk, NY, USA).

## 3. Results

### 3.1. Characteristics

We surveyed 308 HCWs; 137 responded (44% response rate). One-third of respondents had PPE training more than three times. 

### 3.2. Knowledge

Ninety percent of respondents were able to select appropriate levels of respiratory and barrier protection while working with PUI patients. The most important sources of PPE information are the charge nurse (56.7%) and the institutional COVID-19 website (67.3%).

All respondents reported knowledge relevant to safe PPE use and greater than 95% were able to correctly identify when to use hand hygiene (Table 2). More frequent training was associated with greater confidence that the respondent understood how to protect themselves and their patients (Table 2).

### 3.3. Attitude

Sixty-six percent of respondents felt the use of PPE would keep HCWs from contracting COVID-19 in the workplace, and 52.4% reported that PPE would prevent patients from contracting COVID-19 (Table 2). Approximately half (47.6%) of respondents felt that PPE was inconvenient and 28.2% reported that PPE interfered with patient care. Fifteen percent of respondents are willing to care for PUIs without recommended PPE. 

### 3.4. Organizational Factors

Most respondents reported that PPE is readily available (77%) and had knowledge of patients on PPE precautions (80.6%). Over 70% of nurses responded that they would be reprimanded by a supervisor if not using PPE (70.4%), while fewer providers (47%) reported similar perceptions (*p* = 0.007). 

## 4. Discussion

Our survey demonstrated that HCWs employed in two urban EDs have adequate knowledge, a high level of self-reported compliance and adequate access to PPE. However, one-third of respondents do not believe that PPE will protect them from COVID-19, and just under half believe that PPE will not protect patients from COVID-19. This research was conducted prior to vaccine availability.

Self-reported knowledge, attitudes, and organizational factors were similar across nurses and providers, with the exception that RNs stated they would be reprimanded by supervisors if not using PPE more frequently than providers. This may be related to cultural differences between the groups, and/or having a supervisor present during clinical shifts.

Previous studies have suggested that inconvenience is a predictor of poor adherence [2,14,15]. We learned that many respondents found PPE to be inconvenient and believed that PPE interferes with patient care. This indicates a continued need for innovation and enhanced industrial PPE design.

Our health care system is unique as we maintain a high level of pre-existing preparedness for infectious outbreaks, including frequent PPE training prior to the COVID-19 pandemic [16]. Respondents who have completed PPE training more frequently reported increased understanding of how to protect themselves and patients. Respondents reported a frequently updated institutional website with a concise standard operating procedure was the most important source of PPE information. EDs should consider repeat PPE training, and leveraging an institutional website to maintain a high level of preparedness.

The major limitation of this study is the sample size and social desirability bias due to reliance on self-reporting. This survey was administered in two academic EDs with pre-existing experience with infectious diseases so the results may not be generalizable to other EDs. This study also utilized a survey used in previous health care settings but is not a validated instrument. Finally, it should be noted that this study includes several exploratory analyses, thereby increasing the chance of a false positive.

## 5. Conclusions

This cross-sectional survey conducted prior to COVID-19 vaccine availability suggested repeated training on PPE for HCWs may be key in maintaining a high level of confidence among HCWs to safely care for patients and protect themselves, their families and our communities. EDs should consider multiple communication strategies, including a website with concise information and enhanced training for key personnel, in particular the charge nurse. In this study, few HCWs were willing to care for patients without PPE; thus, a continued focus on the availability and accessibility of PPE is paramount. This knowledge can be leveraged by other EDs to help design and implement effective PPE training and communication strategies.

## Figures and Tables

**Table 1:** Characteristics of respondents of personal protective equipment survey in two urban emergency departments during 2020 COVID-19 pandemic.

	RN	Provider	Total
Age (Median, IQR)	31 (26–47)	38 (33–49)	35 (30–48)
Years in practice, % (*n*)			
1–2	27.8% (15)	16.3% (8)	22.3% (23)
3–5	27.8% (15)	24.5% (12)	26.2% (27)
6–10	9.3% (5)	20.4% (10)	14.6% (15)
>10	35.1% (19)	38.8% (19)	36.9% (38)
Number of PPE trainings, % (*n*)			
0–3	63% (34)	71.4% (35)	67% (69)
4–6	25.9% (14)	22.4% (11)	24.3% (25)
7–9	9.3% (5)	0% (0)	4.9% (5)
≥10	1.9% (1)	6.1% (3)	3.9% (4)
Cared for PUI, % (*n*)	92.6% (50)	100% (49)	96.1% (99)
PPE training prior to caring for PUI, % (*n*)	92.6% (50)	98% (48)	95.1% (98)
Had confirmed/suspected COVID-19 infection, % (*n*)	18.5% (10)	14.3% (7)	16.5% (17)

PPE, personal protective equipment; PUI, person under investigation for COVID-19.

**Table 2:** Use of PPE during 2020 COVID-19 pandemic: knowledge, attitudes, and organizational factors with odds ratios.

	RN	Provider	Total	Self-Reported High Compliance Odds Ratio (95% CI) †	Times Received PPE Training Odds Ratio (95% CI) †
Knowledge, % agree or strongly agree (*n*)					
I understand the relevant knowledge of safe PPE use	100% (54)	100% (49)	100% (103)	1.0 (0.97–1.03)	1.51 (0.83–2.72)
I understand the risks of COVID-19 to patients and HCWs	96.3% (52)	91.9% (45)	94.1% (97)	1.02 (0.99–1.05)	1.75 (0.90–3.40)
I understand how to protect myself and patients during COVID-19	98.2% (53)	83.7% (41)	91.2% (94)	1.02 (0.99–1.05)	1.70 (1.0003–2.90)
Identifies when to correctly use hand hygiene	98.1% (53)	91.8% (45)	95.1% (98)	1.0 (0.97–1.03)	1.42 (0.79–2.55)
The correct use of PPE eliminates the need for hand hygiene	1.9% (1)	0% (0)	1% (1)	1.01 (0.97–1.05)	1.11 (0.62–2.0)
Attitude, % agree or strongly agree (*n*)					
Use of PPE and keep HCWs from getting COVID-19	68.4% (35)	67.4% (33)	66% (68)	1.01 (0.99–1.04)	1.30 (0.81–2.11)
Use of PPE will keep patients from getting COVID-19	48.2% (26)	57.2% (28)	52.4% (54)	1.01 (0.98–1.03)	0.76 (0.47–1.23)
PPE use is inconvenient	42.6% (23)	53.1%(26)	47.6% (49)	0.98 (0.95–1.01)	0.66 (0.42–1.05)
PPE use interferes with patient care	24.8% (15)	28.6% (14)	28.2% (29)	0.98 (0.95–1.01)	0.83 (0.52–1.32)
Willing to care for COVID-19 patients	87% (47)	100% (49)	93.2% (96)	0.82 (0.62–1.09)	0.80 (0.32–2.01)
Willing to care for COVID-19 patients w/o the recommended PPE	16.7% (9)	14.3% (7)	15.5% (16)	0.98 (0.95–1.02)	1.17 (0.60–2.30)
More likely to care for patient with COVID-19 if I had a test which told me I had previously recovered from infection	57.4% (31)	55.1% (27)	56.3% (58)	1.01 (0.98–1.05)	0.56 (0.32–0.97)
Organizational factors, % agree or strongly agree (*n*)					
PPE is readily available in the ED	77.8% (42)	77.5% (38)	77.6% (80)	1.01 (0.98–1.04)	1.49 (0.89–2.48)
* Reprimanded by supervisor if not using PPE	70.4% (38)	47% (23)	66.3% (61)	1.02 (0.99–1.05)	1.09 (0.68–1.74)
Knowledge of patients on PPE precautions	77.8% (42)	83.7%(41)	80.6% (83)	0.99 (0.96–1.03)	1.54 (0.93–2.55)
My colleagues often forget to use PPE during patient care	13% (7)	18.4% (9)	15.5% (16)	0.99 (0.96–1.02)	1.20 (0.74–1.94)
I remove PPE immediately after leaving patient room	90.8% (49)	93.9% (46)	92.2% (95)	1.01 (0.98–1.04)	1.21 (0.71–2.05)
I often forget to change PPE between patients	13% (7)	18.3% (9)	15.5% (16)	0.96 (0.93–0.99)	0.78 (0.47–1.27)
Self-reported compliance, M (IQR)	97 (90–100)	95 (90–100)	96 (90–100)		

PPE, personal protective equipment; HCWs, health care workers; ED, emergency department. * Providers vs. nurses (*p* = 0.007). † Odds ratios and 95% CIs evaluate the association between compliance/training and the survey items. Odds ratios greater than 1 indicate that more compliance or training is associated with more of the self-reported knowledge/attitudes/behavior/factors and vice versa.

## Data Availability

Data are stored in a de-identified state and can be made available by reasonable and appropriate request.

## Supplementary Materials

The following are available online at https://www.mdpi.com/article/10.3390/ijerph18137076/s1, PPE Survey.
