# Supplementary material for: Self-Reported Use of Personal Protective Equipment among Emergency Department Nurses, Physicians and Advanced Practice Providers during the 2020 COVID-19 Pandemic
Source: Int J Environ Res Public Health. 2021 Jul 2;18(13):7076. doi: 10.3390/ijerph18137076 (PMC8297270; doi:10.3390/ijerph18137076)
Supplement: Supplementary file 1 [file ijerph-18-07076-s001.zip › ijerph-1219631-supplementary.pdf]

PPE COVID Survey (EUH and EUHM) V2

Consent and HIPAA Authorization Information Sheet

SELF-REPORTED USE OF PERSONAL PROTECTIVE EQUIPMENT AMONG NURSES AND PROVIDERS WORKING IN THE EMERGNECY DEPARTMENT DURING COVID-19 PANDEMIC

Principal Investigator:
Roslyn M Seitz, ENP-C, FNP-C, MPH
Department of Emergency Medicine - Emory Healthcare
Roslyn.Seitz@emoryhealthcare.org
Affiliations: None

Introduction and Study Overview
Thank you for your interest in our survey research study. We would like to tell you everything you need to think about before you decide whether or not to join the study. It is entirely your choice. If you decide to take part, you can change your mind later on and withdraw from the research study.

The purpose of this study is to describe the self-reported use of personal protective equipment among nurses and providers working in the emergency department during the COVID-19 pandemic. This study will take about 10 minutes to complete.

If you join, you will be asked to take a 10 minutes survey now and again in 1-4 months based on emergency department patient volumes.

The potential risks from this study are the loss of time spend taking the survey. You will be asked questions about your use and emergency department use of personal questions that make cause you to feel uncomfortable.

This study is not intended to benefit you directly, but we hope this research will benefit people in the future.

Your privacy is very important to us. There is a law that protects your health information kept by your medical provider; this law is called HIPAA. There are no professional implications to choosing not to complete or participate in the survey. The last 4 digits of phone number are to link surveys over time and will not be used to identify individual participants.

You may revoke your authorization at any time by calling the Principal Investigator, Roslyn Seitz NP, or by writing to the address listed on the information sheet that we will send to you. If identifiers (like your age or the last 4 digits of your phone number) are removed from your PHI, then the remaining information will not be subject to the Privacy Rules. This means that the information may be used or disclosed with other people or organizations, and/or for other purposes.

De-identified data from this study (data that has been stripped of all information that can identify you) may be placed into public databases where, in addition to having no direct identifiers, researchers will need to sign data use agreements before accessing the data. We will remove or code any personal

information that could identify you before your information is shared. This will ensure that, by current scientific standards and known methods, it is extremely unlikely that anyone would be able to identify you from the information we share. Despite these measures, we cannot guarantee anonymity of your personal data.

Contact Information
If you have questions about this study, your part in it, your rights as a research participant, or if you have questions, concerns or complaints about the research you may contact the following:
Roslyn M Seitz, ENP-C, FNP-C, MPH
Department of Emergency Medicine - Emory Healthcare 404-712-7109
or Emory Institutional Review Board: 404-712-0720 or toll-free at 877-503-9797 or by email at irb@emory.edu

Do you have any questions about anything written above? Were there any parts that seemed unclear? If yes, please contact:
Roslyn M Seitz, ENP-C, FNP-C, MPH
Department of Emergency Medicine - Emory Healthcare 404-712-7109
or Emory Institutional Review Board: 404-712-0720 or toll-free at 877-503-9797 or by email at irb@emory.edu

Do you agree to take part in the study?

If Yes please proceed to survey
If No please close browser window

PPE COVID Survey (EUH and EUHM) V2

1. I am a:

- ◯ RN
- ◯ Fellow
- ◯ Attending Physician
- ◯ NP
- ◯ PA
- ◯ Other (survey ends)
- ◯ I prefer not to answer

PPE COVID Survey (EUH and EUHM) V2

2. Years in Practice:

- ◯ 1-2
- ◯ 3-5
- ◯ 6-10
- ◯ 11-15
- ◯ 16-20
- ◯ >20
- ◯ I prefer not to answer

3. During the COVID-19 pandemic I worked primarily at

- ◯ Emory University Hospital (EUH) Emergency Department
- ◯ Emory University Hospital Midtown (EUHM) Emergency Department

4. Historically, I work primarily in:

- ◯ Emergency Medicine
- ◯ Other (please specify)

[ ]

5. Last 4 digits of your cell phone number

[ ]

6. Age

[ ]

7. Have you cared for patients with suspected or confirmed COVID 19 infection?

- ◯ Yes
- ◯ No

8. If yes, did you have training on the use of PPE (ACE, DICE) prior to caring for patients with suspected or confirmed of COVID-19 infection?

- ◯ Yes
- ◯ No

Answer the following questions if you received PPE training (High level, ACE, DICE, CAPR, PAPR)

9. In my training as a healthcare professional, I have received training on PPE (High level, ACE, DICE, CAPR, PAPR) approximately how many times?

- 0-3
- 4-6
- 7-9
- >=10

10. PPE is allocated fairly across personnel involved in patient care and other supporting roles in my work environment.

- Strongly agree
- Agree
- Neither agree nor disagree
- Disagree
- Strongly disagree

Knowledge

11. I understand the relevant knowledge of safe PPE use.

- Strongly agree
- Agree
- Neither agree nor disagree
- Disagree
- Strongly disagree

12. I am confident I understand the risks of the COVID-19 pandemic to patients and healthcare workers.

- Strongly agree
- Agree
- Neither agree nor disagree
- Disagree
- Strongly disagree

13. I am confident I understand how to protect myself and patients during COVID-19 pandemic.

- Strongly agree
- Agree
- Neither agree nor disagree
- Disagree
- Strongly disagree

14. The correct personal protective equipment (PPE) for COVID -19 patients (suspected or confirmed) includes (multiple choices are allowed)

- [ ] Surgical mask
- [ ] N95 mask
- [ ] Gown
- [ ] Gloves
- [ ] Goggles
- [ ] Face shield
- [ ] Hand hygiene
- [ ] CAPR
- [ ] PAPR
- [ ] None of above

15. Hand hygiene includes either washing hands with soap and water, or the use of an alcohol-based hand rub.

- ○ Strongly agree
- ○ Agree
- ○ Neither agree nor disagree
- ○ Disagree
- ○ Strongly disagree

16. I wash my hands with an alcohol-based hand rub when they are visibly soiled.

- ○ Strongly agree
- ○ Agree
- ○ Neither agree nor disagree
- ○ Disagree
- ○ Strongly disagree

17. The correct use of PPE eliminates the need for hand hygiene.

- ○ Strongly agree
- ○ Agree
- ○ Neither agree nor disagree
- ○ Disagree
- ○ Strongly disagree

18. When should you wear an eye protection (i.e. goggles or a face shield), respiratory protection (n95, CAPR or PAPR), gloves and a clean, non-sterile, long-sleeved gown during care for patients with COVID-19? (multiple choices are allowed)

- [ ] During entire treatment and/or nursing care
- [ ] When performing aerosol-generating procedures associated with an increased risk of infection transmission
- [ ] During activities that are likely to generate splashes or sprays of blood, body fluids, secretions, and excretions
- [ ] When performing aspirating or open suctioning of the lower respiratory tract
- [ ] When performing endotracheal intubation
- [ ] When performing CPR
- [ ] When performing bronchoscopy
- [ ] Other (please specify)

19. When should you wear a surgical mask or N95 mask during care for patients with suspected or confirmed COVID-19? (multiple choices are allowed)

- [ ] During entire treatment and/or nursing care
- [ ] If you are working within approximately 1 meter of a patient with suspected or confirmed COVID-19
- [ ] When the patient is on droplet precautions
- [ ] When the patient is on COVID -19 infection precautions
- [ ] Other (please specify)

20. I get information regarding updates for PPE best practice from (multiple choices allowed)

- [ ] Charge Nurse
- [ ] Emory COVID-19 Website
- [ ] Facebook or other social media
- [ ] The news
- [ ] Word of mouth from co-workers
- [ ] Other (please specify)

Attitude

21. The use of PPE will keep healthcare workers from getting COVID-19 infection

- Strongly agree
- Agree
- Neither agree nor disagree
- Disagree
- Strongly disagree

22. Use of PPE will keep patients from getting COVID-19 infection

- Strongly agree
- Agree
- Neither agree nor disagree
- Disagree
- Strongly disagree

23. It is inconvenient to use recommended PPE when taking care for patients with COVID-19 infection

- Strongly agree
- Agree
- Neither agree nor disagree
- Disagree
- Strongly disagree

24. Use of recommended PPE interferes with patient treatment and/or nursing care of patients with suspected or confirmed COVID-19 infection

- Strongly agree
- Agree
- Neither agree nor disagree
- Disagree
- Strongly disagree

25. Are you willing to treat and/or care for patients with COVID-19 infection if you have the opportunity?

- Yes
- No

26. If you choose “no” to the above question, what is the major reason? (multiple choices are allowed)

- [ ] Concern about the possible infection of yourself
- [ ] Concern about the possible infection of your family members
- [ ] Not applicable
- [ ] Other (please specify)

27. Are you willing to treat and/or care for patients with COVID-19 infection if you do not have the recommended (n95 or surgical mask, gown, gloves, face shield) PPE?

- ○ Yes
- ○ No

28. Have you had confirmed or suspected COVID-19 infection?

- ○ Yes
- ○ No

29. I would be more likely to care for patients with suspected or confirmed COVID-19 infection if I had a test which told me if I had previously recovered from the infection (example antibody test)

- ○ Yes
- ○ No

Behaviors and Management

30. All recommended PPE is readily available in the Emergency Department

- ○ Strongly agree
- ○ Agree
- ○ Neither agree nor disagree
- ○ Disagree
- ○ Strongly disagree

31. The charge nurse or other ED staff (RN, Providers) would reprimand me if I did not use PPE when caring for patients with suspected or confirmed COVID-19 infection

- Strongly agree
- Agree
- Neither agree nor disagree
- Disagree
- Strongly disagree

32. I know when patients are on COVID-19 precautions

- Strongly agree
- Agree
- Neither agree nor disagree
- Disagree
- Strongly disagree

33. My colleagues often forget to use recommended PPE when taking care of patients with suspected or confirmed COVID-19 infection

- Strongly agree
- Agree
- Neither agree nor disagree
- Disagree
- Strongly disagree

34. I estimate my compliance to recommended PPE during treatment and/or care of patients with suspected or confirmed COVID-19 infection is (percentage):

0 100

35. I remove gown and gloves immediately when I leave the patient's room

- Strongly agree
- Agree
- Neither agree nor disagree
- Disagree
- Strongly disagree

36. It is easy to forget to change appropriate PPE between patients with suspected or confirmed COVID-19 infection

- ◯ Strongly agree
- ◯ Agree
- ◯ Neither agree nor disagree
- ◯ Disagree
- ◯ Strongly disagree

37. Do you have any suggestions about how to improve PPE use? (free text)

Adapted from: Hu X, Zhang Z, Li N, et al. Self-reported use of personal protective equipment among Chinese critical care clinicians during 2009 H1N1 influenza pandemic. PLoS One. 2012;7(9):e44723. doi:10.1371/journal.pone.0044723
